# Discussing end-of-life issues with terminally ill cancer patients and their families: our results

**DOI:** 10.1186/cc9939

**Published:** 2011-03-11

**Authors:** BE Eftimova, B Lazarova

**Affiliations:** 1 Clinical Hospital, Stip, Macedonia

## Introduction

Most of the literature regarding communication between health professionals and patients at the end of life and their families has focused on specific topics, like breaking bad news and discussing treatment decisions such as CPR and advanced directives. Conversation about end-of-life issues often takes place over time rather than as a single discussion. The objective of this paper is to explore the optimal content and phrasing of information when discussing the dying process and E-O-L issues with terminally ill cancer patients and their families.

## Methods

We conducted focus groups and individual interviews with 20 palliative care patients and their families treated in Clinical Hospital Stip in the past 12 months. The focus groups and individual interviews were fully transcribed. Further individual interviews were conducted until no additional topics were raised. Participant's narratives were analyzed using qualitative methodology.

## Results

Distinct content areas emerged for discussing E-O-L issues: treatment decisions at the E-O-L; potential future symptoms; preferences for place of death; the process of dying; what needs to be done immediately after death; and existential issues. When discussing process of dying participants are recommended: exploring the person's fears about dying; describing the final days and unconscious period; and the reduced need for food and drinks. Many participants identified the dilemma regarding whether to discuss potential complications around the time of death.

## Conclusions

This paper provides strategies, phrases and words that may inform about the process of dying and E-O-L issues. This will be useful especially for patients' families. Further research is needed to determine the generality of these findings.
